# Epithelial Chloride and Bicarbonate Transport in Intestinal Barrier Failure: A Molecular Target-Validation Assessment of CFTR and SLC26A3/DRA in Inflammatory Bowel Disease

**DOI:** 10.3390/ijms27146356

**Published:** 2026-07-17

**Authors:** Yohan Seo

**Affiliations:** Department of Physiology, College of Medicine, Dongguk University, Gyeongju 38066, Republic of Korea; ddukdae12@dongguk.ac.kr; Tel.: +82-54-770-2416

**Keywords:** inflammatory bowel disease, epithelial barrier, SLC26A3/DRA, CFTR, bicarbonate secretion, mucus, anion transport, target validation

## Abstract

Current inflammatory bowel disease (IBD) therapies suppress immune pathways, yet epithelial recovery can remain incomplete. This review evaluates whether intestinal chloride and bicarbonate transport can support a distinct, adjunctive pharmacological strategy. Rather than cataloguing transport proteins, we compare the cystic fibrosis transmembrane conductance regulator (CFTR), SLC26A3/down-regulated in adenoma (DRA), and TMEM16A/ANO1 against an evidence hierarchy of human disease relevance, causal epithelial biology, pharmacological tractability, target engagement, functional rescue, and developability. CFTR and DRA form the most coherent module linking bicarbonate availability to mucin expansion, epithelial surface pH, fluid balance, and barrier organization, but their liabilities differ. CFTR is structurally and clinically druggable, yet its modulators are genotype-directed, and broad activation may worsen diarrhea. DRA has stronger evidence for a colonic barrier role and emerging support from human organoids, but no validated activator or stabilizer. TMEM16A has abundant chemical tools, yet uncertain selectivity, wide extra-epithelial expression, and no established disease-modifying role in IBD. No intervention has achieved mucosal healing through anion-transport rescue in IBD. We therefore define the decisive experiments required before translation: confirmation of persistent functional defects in human tissue, selective exposure-linked rescue in patient-derived epithelium, direct target engagement, and protection against hypersecretion or electrolyte imbalance. The evidence supports focused, mechanism-based evaluation of the CFTR-DRA axis rather than empirical repurposing.

## 1. Introduction: A Barrier-Directed Question for Pharmacology

Inflammatory bowel disease (IBD) treatment has moved from symptom control toward endoscopic, histological, and increasingly barrier-level healing. Biologics and small-molecule immunomodulators have expanded therapeutic options, but primary non-response, secondary loss of response, and incomplete epithelial recovery remain common clinical problems [1,2,3,4]. This residual gap does not imply that inflammation is unimportant. It asks a narrower question: after immune activity is controlled, are there epithelial defects that remain pharmacologically actionable?

Barrier failure in IBD is multifactorial. It reflects interacting epithelial, immune, microbial, and mucus-related processes rather than a single epithelial defect, and ulcerative colitis in particular arises from combined genetic, immune, environmental, and microbial influences. For this reason, the present review does not propose epithelial anion transport as a standalone cause of, or treatment for, IBD. Instead, it asks a narrower and testable question: whether CFTR- and DRA-dependent chloride and bicarbonate transport defines a barrier-relevant epithelial mechanism whose disruption may contribute to disease and whose pharmacological modulation is worth validating in human tissue and patient-derived epithelial models.

The intestinal epithelium is a dynamic interface rather than an inert wall. It couples vectorial ion movement to surface hydration, mucus expansion, luminal pH, tight-junction organization, restitution, and microbial segregation [5,6,7]. Chloride and bicarbonate handling are especially relevant because they determine both fluid movement and the physicochemical conditions under which secreted mucins expand. These functions create an attractive therapeutic concept, but they also create a narrow safety margin: a compound that increases bicarbonate delivery may improve mucus properties yet simultaneously aggravate diarrhea.

Recent reviews have summarized epithelial transport in colitis and the interaction between luminal anion transporters, inflammation, and the microbiome [8,9,10]. The present article has a different purpose. It asks whether individual transport proteins satisfy the requirements of a pharmacological target in IBD. The analysis is therefore organized around evidence strength, tractability, target engagement, functional rescue, and failure modes. This distinction is important because altered expression, structural information, or availability of a tool inhibitor does not by itself establish a therapeutic target.

The scope of this review is deliberately constrained. CFTR and SLC26A3/DRA constitute the most plausible epithelial chloride–bicarbonate module for further validation, whereas TMEM16A/ANO1 is a comparator with context-dependent relevance rather than a general barrier-restorative target. Direct target-specific efficacy in IBD has not yet been shown. The immediate objective should therefore be reducing translational uncertainty in human epithelial systems, not broad clinical repurposing.

## 2. Review Approach and Evidence Hierarchy

This critical narrative review was updated through a focused search of PubMed/MEDLINE and reference lists through 20 June 2026. Search combinations included “inflammatory bowel disease”, “ulcerative colitis”, “Crohn disease”, “CFTR”, “SLC26A3”, “DRA”, “TMEM16A”, “ANO1”, “chloride”, “bicarbonate”, “mucus”, “surface pH”, “organoid”, “epithelial monolayer”, “pharmacology”, “inhibitor”, “activator”, “target engagement”, and “gut restricted”. Priority was given to human mucosal studies, patient-derived epithelial models, causal genetic or loss-of-function studies, pharmacological perturbation with selectivity controls, and work linking transport to barrier-relevant function. Foundational structural and physiological studies were retained when they were necessary to interpret druggability. Full search terms and study selection criteria are listed in Table S1 (Supplementary Materials).

The evidence hierarchy separates six questions. First, is the target altered or functionally abnormal in human IBD? Second, does target loss or gain cause an epithelial phenotype relevant to barrier failure? Third, is the direction of therapeutic modulation defined? Fourth, can engagement be measured directly rather than inferred from transcript abundance? Fifth, does selective modulation rescue mucus, pH, permeability, or restitution in a disease-relevant model? Sixth, can the desired effect be achieved with an exposure profile that avoids secretory, electrolyte, smooth-muscle, airway, vascular, or neuronal liabilities?

This is not a systematic review or meta-analysis, and the evidence domains are not combined into a numerical score. A qualitative matrix is more appropriate because a human association, a selective ligand, and an organoid rescue experiment are not biologically interchangeable. The absence of a clinical-grade activator, for example, cannot be offset by a high score in structural biology.

The core target set was intentionally narrowed. Intracellular chloride-associated proteins, including CLIC4, were not retained because they are not validated apical transepithelial transport targets and currently lack selective, gut-compatible IBD pharmacology. BEST4/OTOP2 cell states and NHE3/SLC9A3 are relevant to epithelial acid-base physiology, but they are treated as contextual biology rather than parallel targets. This boundary prevents the central CFTR-DRA question from being diluted.

## 3. Mechanistic Basis: Why Anion Transport Can Alter Barrier Function

The reason for examining these transporters in IBD is mechanistic: the question is how they shape the epithelial barrier, not whether modulating them already treats disease. CFTR and DRA control chloride and bicarbonate movement at the apical membrane, and these functions influence mucus expansion, epithelial surface pH, hydration, and the local environment immediately above the epithelium. Reduced transporter activity in inflamed mucosa is therefore best interpreted as a barrier-relevant abnormality that warrants formal target validation, not as evidence that restoring the transporter will by itself treat IBD. Throughout this review, biological relevance, pharmacological tractability, and demonstrated therapeutic efficacy are treated as separate questions; evidence in one category is not considered proof of another.

### 3.1. Mucus Expansion and Surface Chemistry

The colonic barrier depends on an organized MUC2-rich mucus system that separates the dense luminal microbiota from the epithelial surface [11,12,13]. The causal importance of this layer is illustrated by spontaneous colitis in Muc2-deficient mice [14]. Newly secreted mucins leave goblet-cell granules in a compact, calcium-rich state. Bicarbonate raises local pH and chelates calcium, permitting rapid expansion and hydration of the mucin polymer [15].

Experimental intestinal studies demonstrate that CFTR-dependent bicarbonate secretion is required for normal mucus release and mucin unfolding [16,17]. In the small intestine, functional CFTR also participates in the release of anchored mucus after microbial stimulation [18]. These studies establish a strong physiological link, but they do not establish IBD drug efficacy. Most were performed in cystic-fibrosis or animal contexts, and the consequences of increasing CFTR activity in an inflamed human colon remain uncertain.

DRA adds a second layer of control. It exchanges luminal chloride for intracellular bicarbonate and contributes to surface pH and chloride absorption. Recent work distinguishes the functions of DRA and CFTR: DRA is particularly important for steady-state surface pH and mucus expansion, whereas CFTR contributes more strongly to stimulus-evoked anion and fluid secretion [19,20,21]. Thus, CFTR and DRA should not be treated as interchangeable “chloride channels”. Their different transport modes predict different efficacy and safety profiles.

### 3.2. Barrier Organization, Inflammation, and Directionality

Ion transport defects can influence barrier integrity through several non-exclusive routes: altered surface pH can change mucin properties and microbial metabolism; impaired chloride absorption can increase luminal water; epithelial stress can disrupt junctional organization; and altered cell differentiation can redistribute transport capacity among epithelial lineages. Active IBD itself produces major changes in absorptive, secretory, goblet, inflammatory, and progenitor cell states [22,23,24,25]. Consequently, a decrease in bulk mucosal transporter mRNA may reflect loss of a cell population, cytokine-mediated transcriptional repression, altered apical trafficking, or direct functional inhibition.

This distinction determines the direction of therapy. Restoring DRA in a DRA-low absorptive epithelium may improve chloride absorption and bicarbonate exchange, whereas indiscriminate CFTR activation may increase secretion. Conversely, inhibiting TMEM16A might reduce pathological calcium-activated secretion in a selected hypersecretory phenotype but would not necessarily repair mucus or junctions. Any development program must therefore begin with a functional phenotype, not with a list of differentially expressed channels.

These epithelial mechanisms operate within a larger, multifactorial process of barrier failure. Recent work shows that barrier breakdown in ulcerative colitis can begin outside the epithelium: an Aeromonas variant that secretes the pore-forming toxin aerolysin selectively kills colonic tissue-resident macrophages, and this macrophage loss can precede overt epithelial inflammation [26]. The experimental findings support causality in the mouse models, whereas the association identified in patients remains observational and requires independent clinical validation. We interpret this finding cautiously, as one emerging mechanism among several rather than a general explanation of IBD, but it reinforces the view taken here: epithelial anion transport should be treated as one component of a broader barrier-failure network. Any hypothesis centred on CFTR, DRA, or TMEM16A must therefore be evaluated alongside immune- and microbiota-driven mechanisms of disease.

**Figure 1:**
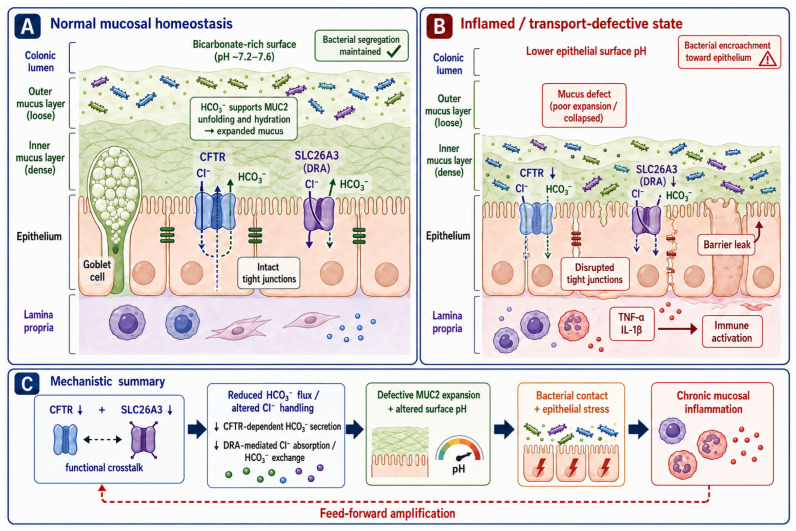
CFTR-SLC26A3/DRA bicarbonate defense in mucus barrier formation. The schematic contrasts normal epithelial bicarbonate availability, mucin expansion, hydration, and microbial separation with an inflamed transport-deficient state. It illustrates a mechanistic hypothesis supported by mucus and transport studies, not proven drug efficacy in IBD [16,17,19,21]. Colored arrows indicate the direction of chloride or bicarbonate movement associated with each transporter. In panel C, solid blue arrows indicate the proposed sequence of barrier deterioration, the black dashed double-headed arrow denotes functional crosstalk between CFTR and DRA, and the red dashed arrow denotes the proposed feed-forward amplification loop. CFTR, cystic fibrosis transmembrane conductance regulator; DRA, down-regulated in adenoma; IL, interleukin; TNF, tumor necrosis factor.

Figure 1 summarizes the mechanistic hypothesis: reduced CFTR-DRA-supported bicarbonate availability can impair mucin expansion, surface pH control, and microbial separation. The figure should be interpreted as a testable causal model, not as evidence that pharmacological activation already improves IBD.

## 4. CFTR: An Established Pharmacology but an Immature IBD Indication

### 4.1. What CFTR Pharmacology Proves

CFTR is an apical, cyclic-AMP-regulated anion channel whose molecular identification, functional characterization, and structural resolution created a benchmark for precision ion-channel pharmacology [27,28,29,30]. Potentiators such as ivacaftor increase gating of responsive variants, whereas corrector combinations improve folding and trafficking of selected mutant proteins [31,32,33]. This body of work proves that epithelial anion transport can be corrected when the molecular defect, direction of modulation, target-engagement assay, and patient selection rule are aligned.

That success cannot be transferred automatically to IBD. Cystic fibrosis modulators were designed for mutation-defined loss of CFTR function. Most patients with IBD do not have a comparable CFTR genotype, and inflamed mucosa can show altered expression without a demonstrated drug-correctable gating or folding defect. One human study reported upregulated CFTR expression in ulcerative colitis, underscoring that transcript abundance alone does not define deficient function [34].

### 4.2. Evidence Relevant to the Intestine

The strongest intestinal rationale for CFTR modulation remains mucus and bicarbonate biology rather than direct IBD efficacy. In people with cystic fibrosis, ivacaftor treatment has been associated with changes in gut microbiota and reduced intestinal inflammatory markers, but that observation occurred in a genetically defined CF population and cannot establish efficacy in IBD [35]. No controlled study has shown that a CFTR potentiator restores mucosal healing in ulcerative colitis or Crohn’s disease.

CFTR target engagement is measurable. Forskolin-induced swelling of intestinal organoids, short-circuit current, and pH-sensitive imaging provide complementary functional readouts [36]. For IBD development, however, swelling alone is insufficient because it primarily reflects net fluid secretion. A candidate should also improve surface pH or mucus expansion and reduce permeability at an exposure that does not produce excessive secretory current.

### 4.3. CFTR Development Considerations

CFTR is therefore best viewed as a pharmacologically validated protein but an unvalidated IBD indication. The most defensible hypothesis is not systemic repurposing of existing modulators. It is local, titratable support of bicarbonate-dependent barrier function in a biomarker-defined subgroup with demonstrably low CFTR-dependent bicarbonate transport or defective mucus expansion. The go/no-go criterion is stringent: barrier rescue must occur at an exposure below that which increases stool water or secretory current. Without that separation, the mechanism is unlikely to be clinically useful.

## 5. SLC26A3/DRA: The Strongest Colon-Specific Rationale but No Suitable Activator

### 5.1. Disease Linkage and Causal Epithelial Biology

DRA is a major apical chloride–bicarbonate exchanger in the distal intestine. Biallelic loss-of-function mutations cause congenital chloride diarrhea, providing unambiguous human validation of its role in intestinal chloride absorption and fluid balance [37,38]. The monogenic phenotype is not IBD, but it defines the physiological consequences of large target perturbation and therefore informs safety. Consistent with a link between reduced DRA function and intestinal inflammation, individuals with congenital chloride diarrhea develop IBD far more often than the general population [39]. This indicates that severe loss of DRA function is compatible with, and may predispose to, chronic colonic inflammation, although it does not by itself show that restoring DRA would be therapeutic.

DRA expression is reduced by intestinal inflammation in experimental models and by TNF-NF-kappaB signaling in epithelial cells [40,41]. More importantly, DRA loss affects more than stool water. Genetic deficiency compromises epithelial barrier integrity, changes junctional organization, and promotes mucosal immune dysregulation [42,43]. Separate studies connect DRA to colonic mucus expansion and surface pH [19,20]. Together, these findings support a causal sequence in which inflammation suppresses DRA, altered chloride–bicarbonate exchange perturbs surface chemistry and absorption, and the resulting epithelial environment reinforces barrier dysfunction. Importantly, the relationship is not purely correlative: in experimental colitis, re-expressing DRA in the epithelium has been reported to preserve tight-junction proteins, improve barrier function, and limit disease severity [44]. Although this was a genetic proof-of-mechanism rather than a drug, it supports the view that DRA is a functional contributor to barrier integrity and a rational, testable target rather than a passive marker of inflammation.

Human-organoid evidence has strengthened this position. In human intestinal epithelium, DRA and CFTR have distinguishable functions: DRA strongly contributes to surface pH regulation, and DRA overexpression can normalize surface pH and MUC2 distribution under conditions of impaired CFTR function, although it does not reproduce CFTR-dependent fluid secretion [21]. This is a meaningful functional rescue experiment, but it was not performed in IBD-derived organoids and did not test a drug.

### 5.2. Pharmacological Tools Reveal a Major Lack of Suitable Compounds

DRA is chemically tractable in the inhibitory direction. DRA_inh_-A250 and related compounds inhibit SLC26A3 from the luminal extracellular surface, reduce colonic fluid absorption, and provide useful mechanistic probes [45,46]. Linaclotide studies further show that DRA can mediate bicarbonate secretion when CFTR function is lost, illustrating functional compensation between transport pathways [47]. These findings validate assayability and ligand access, but inhibition is the opposite of the modulation usually desired for a DRA-low diarrheal barrier phenotype.

No selective DRA activator, positive allosteric modulator, or trafficking stabilizer has entered clinical development. Dexamethasone increases DRA transcription and attenuates DRA loss in experimental colitis, and butyrate can increase Slc26a3 expression through an HDAC8-NF-kappaB-associated pathway [48,49]. Both observations are pharmacologically informative, but neither establishes target-specific DRA therapy. Glucocorticoids and butyrate are pleiotropic; improvement in inflammation cannot be assigned to DRA without genetic or selective pharmacological epistasis.

The priority for medicinal chemistry is therefore unusually clear. A useful program would seek luminally accessible compounds that increase apical DRA abundance, residence time, or exchange activity in DRA-low epithelium. Screening must distinguish direct activation from transcriptional effects caused by broad anti-inflammatory signaling. Counter-screens should include SLC26A6, pendrin, AE2, NHE3, CFTR, and epithelial viability.

### 5.3. DRA Development Considerations

DRA currently has the most coherent IBD-linked epithelial biology among the candidate targets, but the least mature therapeutic chemistry in the required direction. It should be prioritized for targeted validation, not described as clinically ready. The critical proof would be a selective intervention that restores chloride–bicarbonate exchange and apical localization in DRA-low patient-derived epithelium, improves surface pH, mucus, and permeability, and does not create constipation, alkalinization, or electrolyte imbalance.

## 6. TMEM16A/ANO1: A Comparator with Context-Dependent Relevance

TMEM16A/ANO1 is a calcium-activated chloride channel identified independently by expression-cloning approaches [50,51]. Structural studies have defined calcium-dependent activation and pore gating, establishing clear molecular tractability [52,53,54]. Unlike the predominantly epithelial CFTR-DRA module considered here, TMEM16A is distributed across epithelial, smooth-muscle, neuronal, vascular, and interstitial-cell compartments. This breadth creates both opportunities and liabilities.

The pharmacological literature contains several commonly used inhibitors, including T16A_inh_-A01, CaCC_inh_-A01, and MONNA. Their use requires caution because selectivity is assay- and tissue-dependent, and some compounds affect calcium signaling or other conductances [55,56,57]. In intestinal epithelial models, TMEM16A expression and calcium-activated chloride conductance can be increased by growth factor signaling [58]. However, human colonic work has also questioned whether TMEM16A functions as a major apical anion channel, despite evidence that it can modulate CFTR activity [59].

For IBD, neither the direction of modulation nor the target compartment is settled. Inhibition could reduce pathological secretion, urgency, or motility in a selected phenotype, but systemic inhibition risks effects on airway secretion, vascular tone, smooth muscle, and sensory signaling. Activation would create different risks of excessive epithelial secretion. There is no convincing evidence that selective TMEM16A modulation produces mucosal healing in IBD. Accordingly, TMEM16A should not be presented as a comparable target to DRA for barrier restoration. It remains a secondary target for studies in which calcium-activated chloride conductance is directly demonstrated in the relevant epithelial or neuromuscular compartment [60,61].

**Figure 2:**
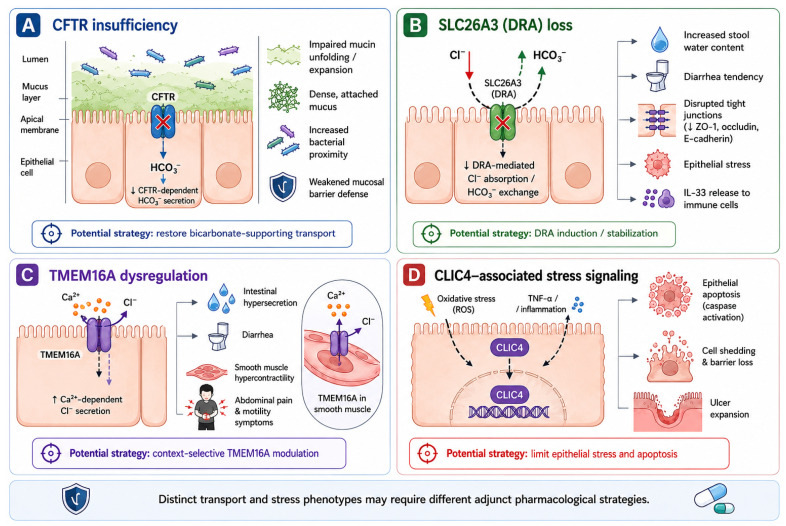
Distinct per-target disease mechanisms and the corresponding logic of pharmacological modulation. (**A**) CFTR insufficiency reduces bicarbonate-dependent mucin unfolding and weakens mucosal defense. (**B**) Loss of SLC26A3/DRA lowers chloride–bicarbonate exchange, raising stool water and destabilizing tight junctions and mucus, with associated epithelial stress and IL-33 release. (**C**) TMEM16A/ANO1 dysregulation drives calcium-activated chloride secretion relevant to hypersecretory, motility, and pain phenotypes, but with broad extra-epithelial expression, including smooth muscle. (**D**) CLIC4 is depicted only as an exploratory intracellular stress and apoptosis node; it is retained for completeness but excluded from the core CFTR-DRA axis. Distinct transport and stress phenotypes would require different, phenotype-matched strategies. Panels illustrate mechanistic hypotheses and are not evidence of drug efficacy in IBD. Arrow colors correspond to the ions or pathways labeled in each panel. Solid arrows indicate ion movement or downstream effects, whereas dashed arrows indicate transport coupling or proposed indirect relationships; neither line style denotes quantitative effect size or evidence strength. CFTR, cystic fibrosis transmembrane conductance regulator; DRA, down-regulated in adenoma; ROS, reactive oxygen species; TNF, tumor necrosis factor.

Figure 2 contrasts the distinct disease mechanisms and the implied direction of pharmacological modulation for each candidate, and situates CLIC4 (panel D) as an excluded exploratory stress node rather than a validated apical transport target.

## 7. Comparative Target Readiness

Table 1 replaces numerical target scoring with a qualitative evidence matrix. The matrix exposes a key asymmetry. CFTR has mature structural pharmacology and clinically validated ligands, but weak IBD-specific target validation. DRA has a stronger colon-specific causal barrier rationale and increasingly informative human epithelial data, but no suitable activator. TMEM16A has multiple pharmacological tools and substantial structural information, but its direction of modulation, selectivity, and relevant tissue compartment remain uncertain. The criteria used to assign the qualitative categories are defined in Note S1 (Supplementary Materials).

This comparison also clarifies why “druggable” and “therapeutically ready” are not synonyms. A target can bind small molecules yet fail because the disease phenotype is absent, the required direction of modulation is chemically inaccessible, or the therapeutic window is too narrow. Conversely, a target can have compelling causal biology but remain undruggable until an appropriate modality and exposure strategy are developed [62].

Table 2 summarizes representative tools and the interpretive limits that should accompany their use. Tool compounds should be treated as instruments for causal inference, not as therapeutic leads unless potency, selectivity, exposure, and tissue context are demonstrated.

## 8. Human Epithelial Models as Target Engagement and Rescue Platforms

### 8.1. Why Transcript Abundance Is Insufficient

The IBD epithelium is remodeled at the levels of cell state composition, chromatin, metabolism, and spatial organization [22,23,25]. Transporter mRNA therefore cannot establish net transepithelial flux. Function also depends on apical localization, phosphorylation, scaffold interactions, electrochemical gradients, and the availability of coupled transport pathways.

Patient-derived intestinal organoids and two-dimensional epithelial monolayers provide a way to separate these variables. Long-term human colonic organoid culture is technically established [63,64], and IBD-derived systems can preserve durable epithelial features, although some inflammatory transcriptional programs normalize during expansion [65,66,67,68]. More recent patient-derived colonoid work shows that pharmacologically reversible epithelial metabolic phenotypes can be identified in ulcerative colitis, supporting the broader value of ex vivo rescue experiments [69].

### 8.2. A Target-Specific Assay Package

For CFTR, target engagement should combine forskolin-induced swelling with CFTR-dependent short-circuit current and bicarbonate-sensitive pH measurements. The IBD-relevant assay package should therefore include mucus expansion or penetrability and permeability rescue at matched exposure.

For DRA, the primary target-engagement assay is chloride–bicarbonate exchange measured by intracellular pH recovery or equivalent flux methods. This should be paired with apical localization, epithelial surface pH, transepithelial resistance, tracer permeability, and mucus organization. Genetic knockdown, CRISPR perturbation, or a selective inhibitor should abolish the candidate drug response; otherwise, the effect cannot be assigned to DRA.

For TMEM16A, calcium-activated chloride current must be measured with simultaneous calcium imaging and pharmacological controls. Because epithelial current may be minor or indirect, organoid or monolayer assays should be complemented by smooth-muscle, neuronal, or interstitial-cell models only when the clinical phenotype implicates motility, urgency, or pain. Assays must include viability and broad ion-channel counter-screens.

Barrier readouts should not be collapsed into a single endpoint. Transepithelial electrical resistance is sensitive to ionic conductance and can be confounded by the same transport changes under study. It should be interpreted together with macromolecular tracer flux, junctional localization, epithelial restitution, and cell death. Human IBD studies have documented disrupted tight-junction architecture and permeability, providing relevant benchmarks for these assays [70,71,72].

### 8.3. Experimental Controls That Determine Credibility

Organoid studies should report donor diagnosis, disease location, inflammatory activity, treatment exposure, sampling from inflamed and non-inflamed regions, passage number, differentiation state, cytokine conditioning and washout, and baseline transport activity. The donor should be the unit of inference; technical replicates cannot substitute for biological replication. Parallel healthy, disease-control, and donor-matched conditions are preferable.

The strongest design is multiple independent perturbations: disease-associated functional deficiency, rescue by a chemically and genetically validated intervention, loss of rescue after target knockdown or antagonism, and recovery of more than one independent barrier readout. This standard is more informative than another expression atlas and directly addresses whether a transport defect is causal, persistent, and drug-responsive.

## 9. Local Exposure, Pharmacokinetics, and Safety

Intestinal transport proteins are attractive targets for local pharmacology because the mucosal surface is accessible from the lumen. Minimally systemic drugs such as tenapanor demonstrate that high intestinal exposure and a low plasma concentration can be engineered for a membrane transporter [73,74,75]. Linaclotide provides a complementary precedent for locally acting epithelial secretory pharmacology [76]. These agents do not validate efficacy in IBD, but they show that gastrointestinal exposure can be designed around a local mechanism.

The desired product profile differs by target. A CFTR-directed compound would require limited, titratable potentiation with explicit ceilings for secretory current and stool water. A DRA-directed compound should increase apical exchange or stability without excessive chloride absorption, constipation, systemic alkalosis, or effects on related transporters. A TMEM16A program would require gut restriction plus counter-screens in airway, vascular, smooth-muscle, and neuronal systems. For all three, luminal concentration alone is inadequate; epithelial tissue exposure, residence time, plasma exposure, and fecal recovery should be related quantitatively to target engagement and response.

Safety should be built into efficacy experiments. Parallel readouts should include epithelial viability, mitochondrial stress, restitution, stool or luminal water and electrolytes, pH, motility, and off-target tissue function. Secretory diarrhea is not a late clinical nuisance but a mechanism-based toxicity that can invalidate the therapeutic concept. The therapeutic window is defined by the separation between barrier rescue and fluid/electrolyte disturbance [77,78].

## 10. Decisive Experiments and Development Priorities

Table 3 defines a staged validation package. The first priority is a human functional map rather than another bulk-expression comparison. Paired biopsies and patient-derived monolayers should quantify CFTR-dependent current, DRA-mediated exchange, surface pH, mucus properties, permeability, and apical localization across active disease, non-inflamed mucosa, remission, and treatment exposure. This will identify whether a stable transport-deficient subgroup exists.

The second priority is selective rescue. For DRA, discovery programs should screen for positive modulation or trafficking stabilization and require target dependence in human epithelial systems. For CFTR, existing modulators may be used as mechanistic probes, but progression should require a bicarbonate/barrier benefit that is separable from net secretion. For TMEM16A, development should stop unless the relevant calcium-activated conductance and target compartment are demonstrated prospectively.

The third priority is mechanistic linkage to inflammation. Barrier rescue may secondarily reduce microbial contact and immune activation, but direct suppression of cytokine pathways should not be assumed. Co-culture or spatially informed models can test whether restored transport changes immune-cell activation, microbial metabolite exposure, or wound-edge behavior. These outcomes should remain secondary until epithelial target engagement is established.

The fourth priority is translational falsifiability. A program should be discontinued if the functional defect disappears after inflammation resolves, if rescue is not target-dependent, if only transcript abundance changes, if the effect is limited to immortalized cell lines, or if barrier improvement occurs only at concentrations that produce secretory or systemic liabilities. These negative criteria protect the field from converting plausible physiology into an unfalsifiable therapeutic narrative.

A conceptual overview that links these priorities to a mechanism-guided, adjunctive workflow is provided as Figure S1 (Supplementary Materials); it is an exploratory research schematic rather than a clinical algorithm and should be read together with the staged criteria in Table 3.

## 11. Conclusions

Epithelial chloride and bicarbonate transport is a credible but unproven pharmacologically relevant component of IBD biology. The evidence is strongest for a CFTR-DRA module that links surface chemistry, mucus organization, absorption, and barrier function. The two targets are complementary rather than equivalent: CFTR offers mature pharmacology but weak IBD-specific validation, whereas DRA offers stronger colonic causal biology but lacks an activator or stabilizer suitable for development.

TMEM16A is structurally tractable but remains a secondary, context-dependent target because its direction of modulation, epithelial contribution, and systemic liabilities are unresolved.

The field should now move from target nomination to targeted validation. A persuasive program must demonstrate a persistent defect in human IBD tissue, direct target engagement, selective rescue in patient-derived epithelium, concordant improvement in surface pH, mucus or permeability, and a therapeutic window that excludes hypersecretion and electrolyte imbalance. Until those conditions are met, epithelial anion-transport modulation should be viewed as an experimentally testable adjunctive strategy, not an established therapeutic class.

## Figures and Tables

**Table 1:** Qualitative evidence matrix for epithelial anion-transport targets in IBD.

Target	IBD-Linked Human Evidence	Causal Barrier Evidence	Pharmacology	Principal Limitation	Current Position
CFTR	Altered expression/function reported, but no reproducible target-defined IBD subgroup or IBD efficacy trial [34]	Strong bicarbonate-mucus mechanism, mainly from cystic-fibrosis and animal systems [16,17]	Approved potentiators/correctors; direct functional assays [31,33,36]	Genotype-directed drugs; broad activation can increase secretion and diarrhea	Mechanistically strong and druggable, but IBD indication remains immature
SLC26A3/DRA	Reduced by inflammation and TNF signaling; emerging human epithelial functional data [21,41]	Loss impairs barrier, mucus expansion, surface pH, and immune homeostasis [19,42,43]	Selective inhibitors validate ligand access; indirect transcriptional up-regulators reported [45,46,48]	No validated activator, stabilizer, or trafficking corrector in the therapeutic direction	Highest priority for focused validation in DRA-low colonic phenotypes
TMEM16A/ANO1	Human IBD evidence is sparse and compartment-specific	Roles in secretion and neuromuscular physiology, but its causal role in the barrier is unresolved [58,59]	Multiple inhibitors and mature structural biology [53,56]	Tool selectivity, uncertain direction of modulation, broad extra-epithelial expression	Secondary target for prospectively demonstrated hypersecretory or motility phenotypes

Evidence categories are not numerically weighted. “Current position” reflects translational readiness, not biological importance.

**Table 2:** Representative pharmacological tools, engagement assays, and interpretive constraints.

Target	Representative Tools	Direct Engagement	Barrier-Relevant Response	Required Controls	Interpretive Constraint
CFTR	Ivacaftor; corrector combinations; forskolin; CFTR_inh_-172	Forskolin-induced swelling; CFTR-dependent short-circuit current; bicarbonate/pH imaging	Mucus expansion, surface pH, tracer flux, restitution	CFTR inhibition or knockdown; matched secretory current; viability	Swelling or current can show secretion without showing barrier repair
SLC26A3/DRA	DRA_inh_-A250/A270/4a as probes; dexamethasone and butyrate as indirect pathway modulators [45,47,48,49]	Cl^−^/HCO_3_^−^ exchange; pH recovery; apical localization	Surface pH, mucus organization, transepithelial electrical resistance (TEER) plus tracer flux	DRA knockdown/inhibition; SLC26 family counter-screen; electrolyte balance	Current chemistry is strongest for inhibition, whereas IBD usually requires restoration
TMEM16A/ANO1	T16A_inh_-A01; CaCC_inh_-A01; MONNA	Ca^2+^-activated Cl^−^current with simultaneous Ca^2+^ imaging	Pathological secretion or phenotype-linked motility/urgency	Orthogonal blockers; genetic perturbation; airway, vascular, neuronal, and smooth-muscle screens	Pharmacological selectivity and relevant tissue compartment are frequently uncertain

Tool compounds are listed for mechanistic use and should not be interpreted as clinically validated IBD therapies.

**Table 3:** Staged validation package and explicit go/no-go criteria.

Stage	Question	Minimum Experiment	Advance Criterion	Stop Criterion
1. Human phenotype	Is a persistent functional defect present?	Paired biopsy/monolayer measurement of flux, surface pH, localization, mucus, and permeability across disease states	Reproducible donor-level defect linked to objective disease context	Only bulk-mRNA change or loss of phenotype after brief resolution of inflammation
2. Target dependence	Is the defect caused by the proposed target?	Genetic perturbation plus selective pharmacological antagonism or rescue	Convergent genetic and pharmacological evidence	Response persists after target loss or is reproduced by unrelated cytotoxic stress
3. Functional rescue	Does modulation repair a relevant epithelial function?	Exposure-response in patient-derived organoids/monolayers using independent barrier readouts	Target engagement precedes and predicts mucus/pH/permeability rescue	Only current or transcript changes; no barrier response
4. Local DMPK	Can intestinal engagement be achieved without systemic exposure?	Luminal, tissue, plasma, and fecal exposure linked to pharmacodynamics	High local exposure, low plasma concentration, reproducible tissue engagement	Systemic exposure required for effect
5. Safety window	Is barrier rescue separated from mechanism-based toxicity?	Parallel stool-water/electrolyte, viability, motility, and off-target tissue assays	Barrier benefit below secretory/electrolyte or extra-intestinal liability threshold	Hypersecretion, constipation, electrolyte imbalance, or tissue toxicity at active exposure

DMPK, drug metabolism and pharmacokinetics.

## Data Availability

No new data were created or analyzed in this study. Data sharing is not applicable to this article.

## Supplementary Materials

The following supporting information can be downloaded at https://www.mdpi.com/article/10.3390/ijms27146356/s1.

## Glossary

**ANO1:** Anoctamin 1; an alternative name for TMEM16A. **Barrier repair**: Restoration of epithelial functions that limit luminal access to the mucosa, including mucus organization, junctional integrity, and restitution. **CFTR**: Cystic fibrosis transmembrane conductance regulator, an apical cAMP-regulated chloride and bicarbonate channel. **DRA**: Down-regulated in adenoma; the SLC26A3 apical chloride–bicarbonate exchanger. **Forskolin-induced swelling**: An organoid assay in which cAMP-dependent CFTR activation drives luminal fluid accumulation and organoid expansion. **Gut-restricted pharmacology**: Drug design that achieves high intestinal exposure with limited systemic absorption. **Functional (pharmacodynamic) rescue**: Exposure-linked correction of a disease-relevant functional phenotype after target engagement. **Target engagement**: Direct evidence that a compound interacts with or functionally modulates its intended target in the relevant tissue. **TMEM16A**: Transmembrane protein 16A, a calcium-activated chloride channel with epithelial and extra-epithelial functions.
