# Supplementary material for: Epithelial Chloride and Bicarbonate Transport in Intestinal Barrier Failure: A Molecular Target-Validation Assessment of CFTR and SLC26A3/DRA in Inflammatory Bowel Disease
Source: Int J Mol Sci. 2026 Jul 17;27(14):6356. doi: 10.3390/ijms27146356 (PMC13409755; doi:10.3390/ijms27146356)
Supplement: Supplementary file 1 [file ijms-27-06356-s001.zip › ijms-4441139-supplementary.pdf]

Supplementary Materials

Epithelial Chloride and Bicarbonate Transport in Intestinal Barrier Failure: A Molecular Target-Validation Assessment of CFTR and SLC26A3/DRA in Inflammatory Bowel Disease

Yohan Seo

Figure S1

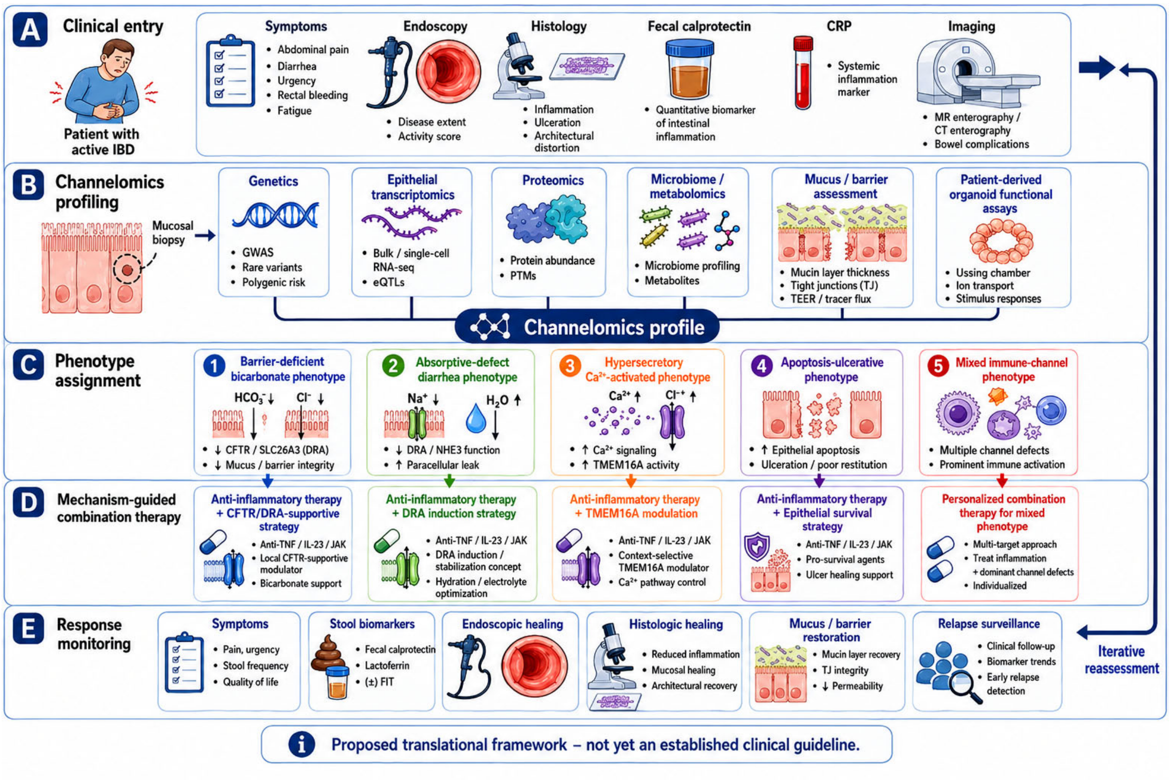

Figure S1. Conceptual translational workflow linking epithelial phenotyping to mechanism-guided, adjunctive pharmacology. Standard IBD assessment is paired with epithelial profiling, patient-derived organoid or monolayer testing, pharmacodynamic readouts, and safety monitoring to assign transport-related phenotypes and, hypothetically, to select adjunctive strategies. This schematic is a proposed research framework and, as indicated within the figure, is not an established clinical guideline; it should be read together with the staged validation criteria in Table 3 of the main text. CFTR, cystic fibrosis transmembrane conductance regulator; CRP, C-reactive protein; DRA, down-regulated in adenoma; FIT, fecal immunochemical test; TEER, transepithelial electrical resistance; TJ, tight junction; TNF, tumor necrosis factor.

Table S1. Literature search strategy and study-selection criteria.

Item	Detail
Review type	Critical narrative review with a focused, scoping-type search; not a systematic review or meta-analysis.
Databases	PubMed/MEDLINE, supplemented by hand-searching of the reference lists of retrieved articles.
Search period	Literature through 20 June 2026.
Disease terms	"inflammatory bowel disease", "ulcerative colitis", "Crohn disease".

Item	Detail
Target terms	"CFTR", "SLC26A3", "DRA", "TMEM16A", "ANO1", "CLIC4".
Function terms	"chloride", "bicarbonate", "mucus", "surface pH", "barrier", "tight junction", "permeability".
Model / pharmacology terms	"organoid", "epithelial monolayer", "Ussing chamber", "pharmacology", "inhibitor", "activator", "target engagement", "gut restricted".
Prioritized evidence	Human mucosal studies; patient-derived epithelial models; causal genetic or loss-of-function studies; pharmacological perturbation with selectivity controls; work linking transport to barrier-relevant function.
Foundational sources retained	Structural and physiological studies included only where necessary to interpret druggability.
Core-target boundary	Intracellular chloride-associated proteins (e.g., CLIC4) and contextual epithelial nodes (BEST4/OTOP2, NHE3/SLC9A3) were treated as background biology, not parallel targets, to keep the CFTR-DRA question undiluted.
Unit of evidence	Where functional claims are made, priority was given to human tissue or patient-derived models over immortalized cell lines.

Search terms were combined with Boolean operators; retrieval was iterative, and citation tracking was used to identify additional primary studies.

Note S1. Definitions of the evidence domains and qualitative categories used in Table 1.

Table 1 of the main text summarizes each candidate target across evidence domains using qualitative categories rather than a numerical score. A numerical composite was deliberately avoided because a human genetic association, a selective chemical ligand, and an organoid rescue experiment are not biologically interchangeable and should not be summed. The domains and categories were applied as follows.

Evidence domains. (i) **IBD-linked human evidence:** human mucosal expression, functional, or genetic data obtained specifically in IBD or in IBD-relevant human tissue. (ii) **Causal barrier evidence:** experimental demonstration that gain or loss of the target produces a barrier-relevant epithelial phenotype, including changes in mucus organization, epithelial surface pH, permeability, junctional integrity, or restitution. (iii) **Pharmacology:** availability of tool or clinical-grade ligands and of direct functional assays, assessed separately for the direction of modulation actually required in IBD. (iv) **Principal limitation:** the single most development-limiting gap for that target. (v) **Current position:** an overall statement of translational readiness.

Qualitative categories. "Strong" denotes convergent, reproducible evidence from human and/or well-controlled experimental systems directly relevant to the domain. "Moderate" denotes supportive but partial, indirect, or context-dependent evidence. "Limited" denotes sparse, preliminary, or compartment-specific evidence. "Absent" denotes no adequate evidence in the relevant direction.

Interpretive rules. Categories reflect translational readiness for IBD, not the intrinsic biological importance of the protein. "Druggable" is not treated as equivalent to "therapeutically ready". Critically, a strong category in one domain does not offset a deficiency in another: for example, mature structural pharmacology for CFTR does not compensate for the absence of a validated activator or stabilizer for SLC26A3/DRA in the therapeutic direction. This asymmetry is the central reason a qualitative matrix, rather than an additive score, was chosen.
